# Sport-related activities as a psychosocial strategy for mental health recovery following natural disasters: a narrative review

**DOI:** 10.3389/fpubh.2026.1894225

**Published:** 2026-07-09

**Authors:** Jiajin Zhou, Jinhu Wei

**Affiliations:** 1 Institute of Physical Education and Health, Yulin Normal University, Yulin, China; 2 College of Physical Education and Health Science, Guangxi Science and Technology Normal University, Laibin, China

**Keywords:** mental health, natural disaster, psychosocial intervention, sport-related activities, trauma recovery

## Abstract

Natural disasters-earthquakes, floods, tsunamis, wildfires, and storms-inflict profound and often enduring psychological trauma on affected populations. Survivors exhibit elevated rates of post-traumatic stress disorder (PTSD), depression, anxiety, peritraumatic dissociation, social dysfunction, and somatic complaints. Despite this burden, mental health services in disaster-affected regions remain under-resourced, particularly in low- and middle-income countries. This narrative review provides a conceptual synthesis of findings from 11 studies that examined the role of sport-related activities in mental health recovery following natural disasters. Recognizing the heterogeneity of the literature, this review proposes a conceptual distinction between “participatory” (e.g., exercise, structured sport) and “spectator-based” (e.g., team identification, media narratives) sport activities, collectively referred to as “sport-related activities.” Through thematic synthesis of quantitative, qualitative, and mixed-methods evidence, the review identifies four interconnected dimensions of post-disaster psychological distress: (a) trauma-specific disorders (PTSD and peritraumatic reactions); (b) affective disorders (depression and generalized anxiety); (c) social dysfunction (isolation, loss of community cohesion); and (d) somatic symptoms (sleep disturbances, pain, fatigue). The review further delineates three proposed mechanisms through which sport-related activities may facilitate recovery: (a) physiological regulation-modulation of the hypothalamic-pituitary-adrenal axis, reduction of cortisol reactivity, and enhancement of neuroplasticity; (b) psychological empowerment-restoration of self-efficacy, meaning-making through narrative reconstruction, and cognitive reframing of traumatic memories; and (c) social reconnection-rebuilding peer support networks, fostering collective identity, and strengthening community resilience. The review concludes that sport-related activities represent a promising, low-cost adjunctive strategy for post-disaster mental health recovery. However, given the methodological heterogeneity and observational nature of the included evidence, these findings cannot establish causal effects. The review serves as a foundational step for future, more rigorous systematic and scoping reviews to build upon.

## 1 Introduction

Natural disasters are among the most devastating and uncontrollable stressors that human societies face. This has resulted in significant human and economic losses for the international community, the 2024 Valencia (Spain) DANA floods caused 223 fatalities, displaced 15,000 residents, and incurred financial losses estimated at over 50 billion (1). In addition, the 2023 Turkey-Syria earthquakes—two massive shocks of magnitudes 7.7 and 7.6—affected 14 million people, caused over 50,000 fatalities, and injured 107,000 individuals (2). The 2011 Great East Japan Earthquake and tsunami resulted in approximately 18,500 deaths and displaced hundreds of thousands (3). These figures underscore the staggering human and economic toll of climate-exacerbated extreme weather events.

In addition to economic and physical losses, natural disasters also have widespread psychological effects. A systematic review and meta-analysis of 76,101 earthquake survivors found that nearly one-quarter (23.7%) met diagnostic criteria for PTSD in the months following the event (4). The prevalence of depression ranged from 3.23% to 52.70% and anxiety from 2.2% to 84% across individual studies included in Keya et al.'s (5) meta-analysis (2023). Beyond clinical disorders, survivors experience peritraumatic dissociation (e.g., derealization, depersonalization, time distortion), sleep disturbances, social withdrawal, and prolonged grief (2, 6). This is because natural disasters can result in physical injury, property damage, forced displacement, and loss of loved ones (6, 7).

Despite this substantial psychological burden, mental health services in disaster-affected regions, particularly low- and middle-income countries, remain severely under-resourced. Shortages of trained mental health professionals, destruction of health infrastructure, displacement, stigma, and economic constraints collectively limit access to evidence-based psychotherapies such as trauma-focused cognitive-behavioral therapy (TF-CBT) and eye movement desensitization and reprocessing (EMDR) (8, 9). At the same time, many disaster victims with physical disabilities face significant challenges in their rehabilitation; unable to access the necessary medical resources, they must rely on community-based rehabilitation to recover (10). Consequently, there is growing interest in alternative, community-based interventions that can be delivered by non-specialists, integrated into existing social structures, and sustained over the long recovery period.

Sport and physical activity have emerged as promising adjunctive interventions in this context. A substantial body of evidence demonstrates that exercise therapy reduces symptoms of PTSD, depression, and anxiety in clinical and non-clinical populations (3). In post-disaster settings specifically, sport participation has been associated with improved psychological well-being, reduced depressive symptoms, and enhanced social connectedness (3, 11, 12). Sport programs have been implemented following earthquakes in Iran, Japan, Italy, Turkey, and New Zealand (3, 6–8, 11, 13, 14); after hurricanes in the United States (15–17); following floods in Brazil and Pakistan (9, 18). However, the mechanisms underlying these effects remain poorly specified, the quality of existing evidence is heterogeneous, and systematic integration across disaster types and cultural contexts is lacking.

This narrative review aims to conceptually synthesize existing research on sport-related activities as a psychosocial strategy for mental health recovery following natural disasters. Specifically, the review addresses three questions: (a) What are the predominant mental health consequences of disaster exposure across different populations and disaster types? (b) What potential pathways and conceptual frameworks have been proposed to explain how sport-related activities may facilitate psychological recovery? and (c) What program characteristics appear to be associated with improved outcomes? By integrating findings from cross-cultural, interdisciplinary studies, the review seeks to identify common themes, inform future research priorities, and provide a conceptual foundation for practitioners and policymakers.

## 2 Methods

### 2.1 Review design

This study employs a narrative review methodology. Narrative reviews are particularly well-suited for synthesizing heterogeneous evidence from diverse disciplines (e.g., clinical psychology, public health, sports science, disaster medicine, sociology) and for presenting findings in an accessible format for non-specialist audiences, including policymakers and humanitarian practitioners (19). In line with this design, our goal is not to exhaustively cover all literature, but to identify common themes, propose a conceptual framework, and highlight gaps for future research.

**Figure 1:**
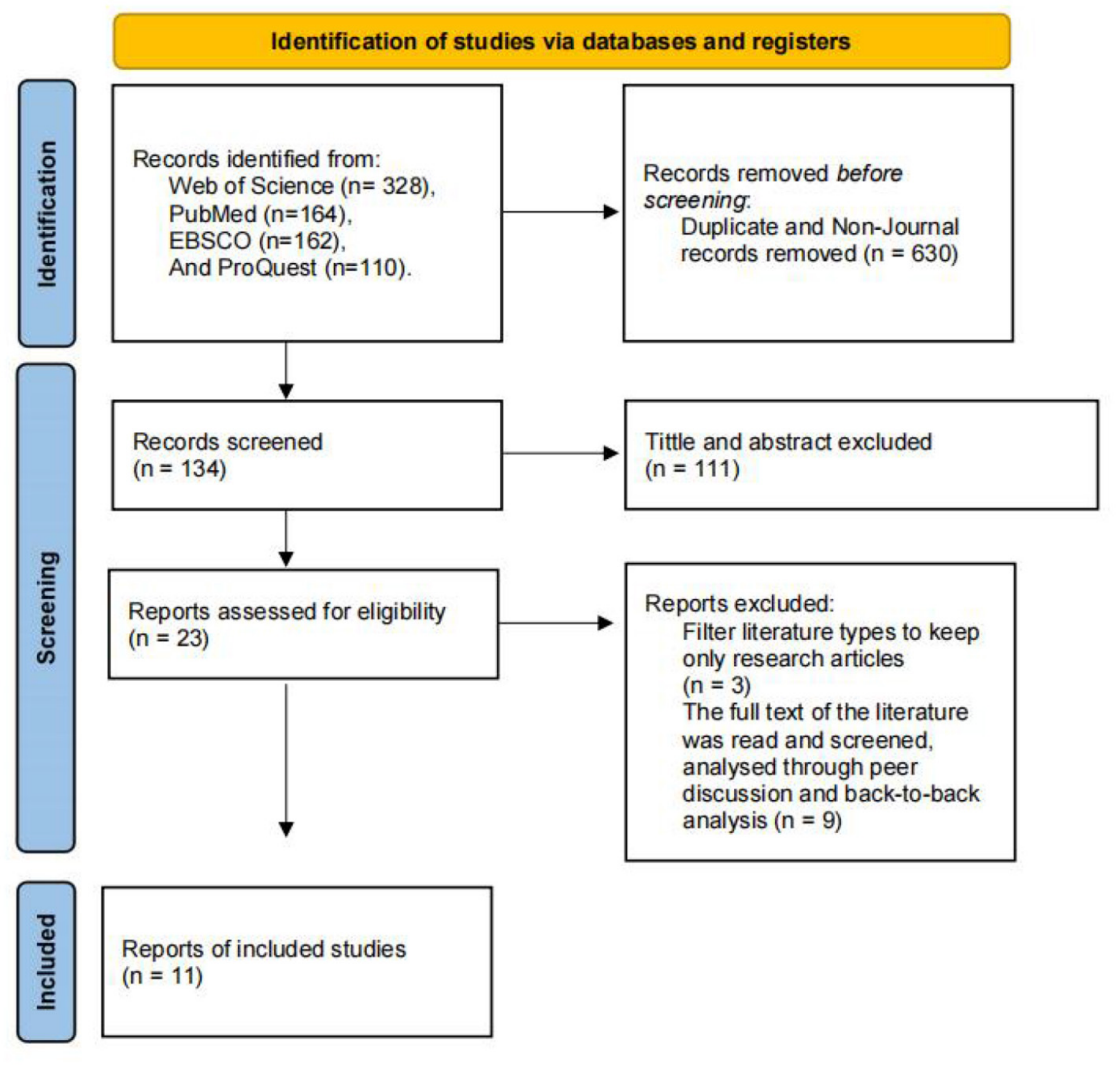
PRISMA 2020 flow diagram for study selection. Adapted from https://www.prisma-statement.org/, licensed under CC BY 4.0.

To enhance methodological transparency, the process of literature search and selection is reported in accordance with the PRISMA 2020 statement (Figure 1), although the analysis remains thematic and interpretive, characteristic of a narrative review.

### 2.2 Search strategy and study selection

A systematic literature search was conducted in Web of Science, then conducted a search using databases such as PubMed, EBSCO, and ProQuest to ensure that no key literature was overlooked (studies published between January 2000 and May 2026). Search terms included combinations of: (“natural disaster” OR “earthquake” OR “flood” OR “hurricane” OR “tsunami” OR “wildfire” OR “cyclone”) AND (“sport” OR “physical activity” OR “exercise” OR “recreation” OR “athletic participation” OR “team identification”) AND (“mental health” OR “PTSD” OR “posttraumatic stress” OR “depression” OR “anxiety” OR “trauma” OR “psychosocial” OR “well-being” OR “resilience”).

Studies were included if they: (a) involved human populations exposed to a natural disaster (earthquake, flood, hurricane/cyclone, tsunami, wildfire, or volcanic eruption); (b) examined sports participation, physical activity, exercise, or team identification as an exposure or intervention; (c) reported quantitative, qualitative, or mixed-methods outcomes related to mental health (e.g., PTSD, depression, anxiety, psychological distress, well-being, resilience, social cohesion); (d) were published in peer-reviewed journals or as book chapters with original data; (e) were written in English. Exclusion criteria were: case reports, editorials/commentaries, conference abstracts without full data, studies of non-disaster trauma (e.g., combat, interpersonal violence), and studies where the disaster exposure was indirect (e.g., media exposure only).

### 2.3 Data extraction and conceptual synthesis

From the initial pool of 134 records, after removing duplicates and applying eligibility criteria, 23 full-text articles were assessed. The final synthesis included 11 studies (see Table 1). Following the thematic synthesis approach described by Thomas and Harden (26), Two authors (J. Z. and J. W.) independently conducted line-by-line coding of the results sections of the included studies. Initial codes were generated inductively from the data. The authors then met to compare and discuss their codes, with disagreements resolved through consensus-building discussions. Through this iterative process, descriptive themes were developed and subsequently refined into the analytical themes that structure the Results and Analysis section.

**Table 1:** Summary of the sports in promoting mental health recovery among victims of natural disasters.

No.	Citation	Study population (*N*)	Disaster type	Role of sport
1	Çakir et al. (6)	*N* = 678 university students (378 males and 300 Female)	2023 Turkey Twin Earthquakes	Regular physical activity was linked to lower trauma (exercisers: 67.71 vs. non-exercisers: 71.97, *p* < 0.001). Among active students, trauma positively correlated with seeking social support (*r* = 0.394, *p* < 0.01) and coping strategies (*r* = 0.176, *p* < 0.01), indicating exercise facilitates adaptive coping and reduces post-earthquake psychological distress.
2	Erlichman and Harrison (17)	*N* = 249, Locals affected by Hurricane Harvey	2017 Houston Hurricane Harvey	Identification with the Houston Astros predicted reflected glory (beta = 0.37, *p* < 0.001). Reflected glory enhanced coping with tragedy (*b* = 0.37, 95% CI [0.26, 0.49]), partially mediated by self-efficacy [indirect effect *b* = 0.06, 95% CI (0.01, 0.14)]. Coping increased meaning in life (beta = 0.37, *p* < 0.001). Thus, the team's World Series win facilitated psychological recovery by boosting self-efficacy, coping, and existential meaning.
3	Fortunato (15)	Case study: Florida Panthers (NHL); key informant interviews with team president et al.	2017 Florida Hurricane Irma	A professional ice hockey team's corporate social responsibility actions—including fundraising, shelter provision, and supply distribution—supported community relief efforts and strengthened community cohesion after Hurricane Irma.
4	Inoue et al. (12)	*N* = 893 spectators of J. League soccer games	2011 Great East Japan Earthquake	Sport teams provided emotional support: team identification predicted perceived emotional support (*b* = 0.31, *p* < 0.001), which enhanced community cohesion (*b* = 0.24, *p* < 0.001), partially mediating identification's effect on well-being [indirect effect = 0.07, 99% CI (0.044, 0.114)]. This highlights sport's capacity to offer psychosocial resources fostering social well-being after disaster.
5	Kunz, 2009 (8)	*N* = 15 parents	2003 Bam Earthquake	A structured sport and play program facilitated children's psychosocial recovery after the Bam earthquake by fostering teamwork and self-confidence, with locally recruited coaches playing a crucial role in providing emotional support and rebuilding social connections.
6	Özdemir et al. (13)	*N* = 389 adolescents (aged 11–17, 48.3% female and 51.7% male)	2023 Kahramanmaraş Earthquakes	Physical activity level was weakly negatively correlated with depression (*r* = −0.228, *p* = 0.012), but not with PTSD (*r* = −0.026, *p* = 0.614) or anxiety (*r* = −0.077, *p* = 0.130). “Physical activity may help alleviate depressive symptoms.” The study recommends early physical activity encouragement and community interventions.
7	Serazio (16)	N/A (Critical analysis of media narratives)	2005 Hurricane Katrina	Media narratives linking a professional football team's success to post-Katrina recovery provided collective coping meaning and temporary respite but risked diverting attention from unresolved material needs.
8	Thorpe (14)	*N* = 14 action sport participants (11 males, 3 females; aged 18–49)	2011 New Zealand Christchurch Earthquake	Participation in recreational action sports helped Christchurch earthquake survivors restore a sense of normalcy, identity, and community reconnection by creatively engaging with the disrupted physical environment.
9	Valenti et al. (7)	*N* = 179 adolescents aged 14–18 (87 boys, 92 girls); *N* = 149 (70 boys and 79 girls)	2009 L'Aquila Earthquake	Sports practice moderated some earthquake effects, notably anxiety (A-anx: quake times sport beta = −3.44, *p* < 0.001), though not depression (*p* = 0.37). Overall, sport was associated with attenuated psychological distress, supporting its role as a coping strategy fostering resilience after disasters. The study recommends integrating sports into adolescent disaster management policies.
10	Adhikari et al. (10)	*N* = 13 age from 9 to 80 (mean ± standard deviation: 50.85 ± 21.72)	2015 Nepal Earthquake	All participants completed the rehabilitation without adverse effects. The treatment demonstrated significant reduction in disability level (*p* < 0.001, effect size = 0.63) and pain level (*p* = 0.007).
11	Zhang et al. (3)	3,567 older survivors (aged 65+)	2011 Great Eastern Japan Earthquake	Greater sports group participation groups was significantly associated with reduced depressive symptoms (*B* = −0.003, Cohen's *f*^2^ = 0.10, *p* = 0.01) after multivariable adjustment. This protective effect did not differ by housing damage severity (interaction *p* = 0.21). Physical activity combined with social engagement may alleviate post-disaster depression through endorphin release, neural growth, and enhanced social support, highlighting the importance of group exercise in disaster recovery.

A key conceptual issue emerged during this process: the need to define and categorize the heterogeneous “sport” construct. Through our inductive analysis and consensus discussions, we found that the 11 studies addressed two distinct forms of engagement: (1) Participatory Sport Activities, which include direct engagement such as sport participation, exercise, physical activity, and recreational activities; and (2) Spectator-based Sport Activities, which include indirect engagement such as team identification, sport fandom, and media consumption of sport. For example, studies addressing team identification (12, 17), sports media narratives (16), and organizational crisis responses (15) were classified as spectator-based sport activities. While mechanistically different, both categories were found to serve as potential psychosocial resources for disaster-affected individuals. Therefore, in this review, we use “sport-related activities” as an overarching conceptual term to encompass both of these empirically identified forms of engagement.

## 3 Results and analysis

### 3.1 Mental health consequences of natural disasters

The included studies consistently documented elevated psychological distress among disaster survivors across multiple domains. Analysis revealed four interconnected dimensions of post-disaster mental health burden: trauma-specific disorders (PTSD and peritraumatic reactions), affective disorders (depression and anxiety), social dysfunction (isolation and loss of community cohesion), and somatic symptoms (sleep disturbances and psychosomatic complaints). These dimensions are not mutually exclusive and frequently co-occur, but they exhibit distinct correlates and may require differentiated intervention approaches.

#### 3.1.1 Trauma-specific disorders resulting from PTSD and peritraumatic

PTSD constitutes the most extensively studied psychological consequence of disaster exposure. Hyland et al. (2) used exploratory structural equation modeling to find that disaster-affected populations face four factors: peritraumatic dissociation, peritraumatic stress, anxiety/depression, and PTSD. In particular, physical injuries sustained by the individuals themselves or their family members were significantly associated with higher levels of PTSD severity (β = 0.13–0.15, *p* < 0.01), and female gender was the strongest predictor (β = 0.26, *p* < 0.001) (2).

Peritraumatic reactions-distress experienced during and immediately after the trauma-are also highly prevalent. Massazza et al. (20) demonstrated that peritraumatic dissociation (e.g., feelings of unreality, “blanking out,” disorientation) and peritraumatic stress (e.g., helplessness, horror, fear of death) predict subsequent PTSD symptoms. In Hyland et al.'s (2) factor analysis, peritraumatic dissociation and peritraumatic stress emerged as distinct but correlated factors (*r* = 0.49), suggesting that these represent separable phenomenological experiences requiring differentiated intervention. Notably, requiring food and water aid was uniquely associated with peritraumatic stress (β = 0.11, *p* = 0.033), while having a family member injured predicted peritraumatic dissociation (β = 0.16, *p* = 0.001) (2).

Similarly, a portion of studies have indicated that conducted a 5-year follow-up of survivors of the 2011 Great East Japan Earthquake and Tsunami, finding persistent mental health impacts, with displaced individuals showing elevated depression and PTSD symptoms even after controlling for baseline health (5, 21).

#### 3.1.2 Affective disorders resulting from depression and generalized anxiety

Depression and anxiety are equally prevalent and may persist for years or decades after the disaster event. Zhang et al. (3) prospectively followed 3,567 older Japanese survivors (aged ≥65 years) from pre-disaster (2010) to post-disaster (2013) using the 15-item Geriatric Depression Scale (GDS). Mean GDS scores increased by 0.13 points (SD = 2.88) over the period, and mild-to-severe depressive symptoms (GDS ≥ 5) were newly identified in 15.3% of those without depressive symptoms at baseline (3). Among survivors, those who experienced housing damage had significantly higher GDS scores (*p* < 0.001) (3).

Valenti et al. (7) conducted a unique before-and-after study of 149 adolescents (aged 14–18) who experienced the 2009 L'Aquila (Italy) earthquake. Using the Minnesota Multiphasic Personality Inventory-Adolescent (MMPI-A), the authors found significant increases in depression (A-dep: mean 47.54 pre-quake vs. 52.67 post-quake, *p* < 0.001), social discomfort (A-sod: 49.91 vs. 51.72, *p* < 0.001), and anxiety (A-anx: 46.73 vs. 54.97) scores (7). Importantly, adolescents who practiced sports regularly (≥2 times/week, ≥1 h/session) showed sports practice significantly moderated anxiety increases (*p* < 0.001), but the moderation effect for depression was not statistically significant (*p* = 0.37). Nonetheless, the general pattern suggested a protective trend (7).

Overall, depression and anxiety triggered by natural disasters are highly likely to lead to emotional disorders among affected populations. Therefore, we need to implement specific interventions to mitigate these issues.

#### 3.1.3 Social dysfunction and community disruption

Disasters systematically disrupt social networks, erode community cohesion, and induce social withdrawal-factors that both exacerbate and are exacerbated by psychological distress. Domínguez and Yeh (22) conducted online questionnaire interviews with 259 survivors of the 2017 Northern California wildfires (the most destructive fires in California history) 3 months after the fires ended. They found that loss and displacement were pervasive: participants reported losing homes, pets, photos, heirlooms, and “all the future opportunities that are now lost to me.” (22). Participants also described exploitation (rent inflation, insurance scams) and pronounced social inequities: “Those who were suffering prior to the fires are suffering worse now… the homeless and undocumented went even more ignored” (22).

#### 3.1.4 Somatic symptoms and sleep disturbances

Somatic complaints, particularly sleep disorders, represent a fourth dimension of post-disaster distress that is often under-recognized in mental health assessments. In Çakir et al.'s (6) study, the “Sleep Problems” subscale of the Post-Earthquake Trauma Level Scale had a Cronbach's α of 0.85, indicating good internal consistency. Female participants reported significantly higher sleep disturbance scores than males (11.86 vs. 11.18, *p* = 0.02), with a small effect size (η^2^ = 0.014) (6). Domínguez and Yeh (22) documented physical symptoms, including respiratory difficulties (requiring evacuation out of state), hair loss, menstrual irregularities, and stress-induced heart attacks. One participant described: “I was taken to the ER 12 h after feeling my first chest pain… I had a stress-induced heart attack and my heart inflamed”.These studies collectively demonstrate that disaster-affected individuals experience somatic symptoms and sleep disturbances in the aftermath of a disaster.

### 3.2 Characteristics associated with improved outcomes

While the included studies are too heterogeneous to allow for definitive conclusions about effectiveness, we identified several program characteristics that appeared to be associated with more favorable outcomes. These patterns should be interpreted as descriptive and exploratory. These findings should be regarded as descriptive and exploratory, and may serve as a reference and guide for future research.

#### 3.2.1 Structured, facilitated programs and unstructured participation

Structured programs delivered by trained facilitators were consistently associated with lower levels of psychological distress in the reviewed studies. Kunz (8) emphasized that “the coaches and their efforts to create a supportive environment play a crucial role in using sport and play as effective instruments for supporting the post-disaster psychosocial rehabilitation process”. In the Bam program, coaches were recruited from the local population (allowing them to “know their situation and share the same experiences”), received ongoing training in sport didactics and psychosocial issues, and were selected based on motivation and interpersonal skills rather than athletic expertise (8). This local recruitment facilitated trust, cultural appropriateness, and sustainability; after the project ended, former coaches continued to run activities through a locally managed sports center (8). These qualitative accounts suggest that facilitation quality is an important contextual factor, but they cannot confirm that structured programs are more effective than unstructured ones.

Conversely, studies examining unstructured or self-directed physical activity showed weaker or null associations. For instance, Özdemir et al. (13) found that physical activity was negatively correlated with depression (*r* = −0.228) but not with PTSD severity (*r* = −0.026, *p* = 0.614), implying that the presence of structured psychosocial support components might partly explain differences in study findings. The absence of structured psychosocial support components (e.g., group reflection, psychoeducation, cognitive restructuring) may account for this discrepancy; however, the observational nature of the evidence prevents firm conclusions.

#### 3.2.2 Group-based and individual exercise

Group-based exercise appears superior to individual exercise for improving social wellbeing and reducing depression. Tsuji et al. (11) found that both group exercise participation (e.g., sports groups, hobby groups) and regular walking were protective against worsening depressive symptoms. However, participation in voluntary groups or senior citizens' clubs did not yield significant benefits after multivariable adjustment, suggesting that the type of group matters: sports and hobby groups (which involve physical activity and shared interests) may be more effective than purely social groups (11). Zhang et al. (3) similarly found that increased participation in sports and hobby groups was associated with reduced depressive symptoms, whereas participation in voluntary groups or senior citizens' clubs was not. These studies suggest that participating in team sports may provide greater mental health benefits for disaster victims, which may be related to the supportive effects of team dynamics in such activities. It should be noted, however, that since these comparisons are derived from different subgroups rather than head-to-head experimental designs, they should be viewed as hypothesis-generating patterns rather than evidence of superiority.

#### 3.2.3 Cultural adaptation

Culturally adapted interventions, particularly those that respect local religious and gender norms, show higher uptake and retention. In the Bam program, gender-segregated classes, a female-only coaching staff, and curtained windows to prevent external viewing enabled over 50% female participation in a conservative context (8). Turkmen (23) surveyed Turkish female university students and found a weak positive correlation between religiosity and sport participation, suggesting that moderate religious beliefs may actually promote physical activity through collective rituals and health-promotion values. This complexity underscores the need for nuanced, context-sensitive adaptation rather than blanket assumptions about religious barriers.

#### 3.2.4 Dose, duration, and timing

The optimal dose and timing of sport interventions remain unclear due to heterogeneous protocols. Interventions ranged from 6 weeks to 12 months (10, 24). Frequency varied from once weekly to daily (8, 10). Session duration ranged from 30 min to 90 min (8, 10).

Despite this heterogeneity, some patterns emerge. Longer-term interventions (≥6 months) appeared to be associated with more sustained improvements in disability and quality of life, yet the absence of control groups makes it impossible to attribute these improvements solely to the intervention. The Bam program's daily sessions over approximately 12 months likely contributed to its observed effects, but the lack of a control group precludes causal attribution (8). Timing also matters. Hyland et al. (2) collected data 43 days post-earthquake and found that peritraumatic dissociation and stress factors were already distinguishable, suggesting that early interventions (within weeks) should target peritraumatic reactions specifically. Domínguez and Yeh (22) surveyed survivors 3 months post-wildfires and documented ongoing need for mental health support, indicating that interventions should extend well beyond the acute emergency phase. In contrast, Valenti et al. (7) conducted assessments approximately 10 months post-earthquake and still detected significant increases in depression, anxiety, and social discomfort, underscoring that the recovery window is measured in months to years.

## 4 Discussion

This narrative review conceptually synthesized evidence from 11 heterogeneous studies examining sport-related activities in post-disaster mental health contexts. Rather than establishing causal effects, the review proposes a conceptual framework that distinguishes between participatory and spectator-based forms of engagement—both of which were associated with psychosocial benefits in the reviewed literature. The four dimensions of psychological distress (trauma-specific disorders, affective disorders, social dysfunction, and somatic symptoms) and the characteristics associated with improved outcomes provide a structured lens for interpreting the current evidence and guiding future inquiry. Based on the themes identified in the Results, we propose three interconnected pathways through which sport-related activities may support recovery. These should be regarded as interpretive syntheses, not as empirically validated mechanisms.

### 4.1 Proposed conceptual framework and mechanisms

Our synthesis identified a set of sport-related activities in the literature that, while heterogeneous, could be organized into two conceptual categories. The first, “participatory sport activities,” involves direct, often physical, engagement, such as community-based rehabilitation exercises (10), structured sport and play programs (8), and regular physical activity or group sports (3, 6, 7, 13, 14). The second, “spectator-based sport activities,” involves indirect, often socio-psychological, engagement, such as team identification (12, 17), corporate social responsibility (CSR) initiatives by professional sport organizations (15), and collective engagement with sports media narratives (16). While these categories operate through different primary pathways—physiological/behavioral vs. socio-cognitive—our analysis suggests they converge on improving mental health by providing resources for physiological regulation, psychological empowerment, and social reconnection. Through an analysis of the 11 included studies, we identified three potential mechanisms through which sport-related activities may be associated with mental health recovery following a disaster. These mechanisms are not mutually exclusive and likely operate synergistically.

#### 4.1.1 Physiological regulation as a proposed pathway

Regular physical activity exerts direct effects on stress-responsive neurobiological systems, including the hypothalamic-pituitary-adrenal axis, autonomic nervous system, and endocannabinoid system. Exercise has been shown to reduce cortisol reactivity to psychosocial stress, and enhance hippocampal neuroplasticity-mechanisms implicated in both stress resilience and recovery from trauma (25). However, these physiological pathways were not directly tested in the disaster-specific studies reviewed here. Thus, they remain plausible hypotheses that require future empirical validation.

In post-disaster contexts, participatory sport interventions have yielded preliminary physiological and physical benefits. For example, physical activity programs can help alleviate somatic pain, thereby potentially improving their sleep quality. Adhikari et al. (10) have shown that physical activity levels of moderate intensity or higher can effectively reduce patients' pain levels (*p* = 0.007). Thordardottir et al. (24) reported that a 6-week hatha yoga program improved sleep, concentration, wellbeing, quality of life, depression, and anxiety among earthquake survivors. At the same times, Çakir et al. (6) found that students who engaged in regular physical activity (≥2 times/week) had significantly lower post-earthquake trauma scores (67.71 vs. 71.97, *p* < 0.001, η^2^ = 0.027) and lower sleep disturbance scores than inactive peers.

#### 4.1.2 Psychological empowerment as a proposed pathway

Engagement with sport-related activities can be associated with the restoration of psychological resources eroded by trauma, including self-efficacy (belief in one's capacity to execute behaviors), mastery (sense of control over outcomes), and meaning-making (integration of traumatic experiences into a coherent narrative).

Meaning-making-the process of transforming traumatic experiences into a narrative of recovery-was prominent in qualitative studies. Domínguez and Yeh (22) documented that survivors used storytelling, art practices, and philosophical dialogues to reconstruct meaning. One participant articulated this transformation: “The trauma of nearly losing our lives… also how it triggered earlier and intergenerational traumas… Our sense of identity is forever altered; also, what truly matters. Our sense of possibility is wounded; also, our sense of belonging. The losses arising out of the fire are staggering… Yet, at the same time, so are the blessings” (22). Sport, as a structured, goal-directed activity with clear feedback (e.g., scoring a goal, completing a set), may facilitate meaning-making by providing concrete evidence of progress and competence in a context where survivors often feel helpless.

Quantitative studies have observed this link. Zhang et al. (3) found that increased frequency of participation in sports groups was associated with reduced depressive symptoms (*B* = −0.003, Cohen's *f* ^2^ = 0.10, *p* = 0.01) after controlling for age, sex, education, ADL impairment, income change, and disaster-related stressors. The effect size was modest but significant, suggesting that sports participation contributes incrementally to psychological recovery beyond the effects of other protective factors (e.g., social support, physical health) (3).

Team identification and reflected glory from spectator-based sport activities also contribute to psychological empowerment. Erlichman and Harrison (17) surveyed 249 Houston residents who were impacted by Hurricane Harvey (August 2017) and who also followed the Houston Astros' first World Series win (November 2017). Using organizational identification theory and basking in reflected glory (BIRGing), they found that stronger organizational identification with the Astros led to stronger reflected glory (β = 0.37, *p* < 0.001), which in turn was associated with better coping with tragedy [direct effect: *b* = 0.37, 95% CI (0.26, 0.49)] (17). Self-efficacy partially mediated this relationship [indirect effect: *b* = 0.06, 95% CI (0.01, 0.14)], and coping predicted greater meaning in life (β = 0.37, *p* < 0.001) (17). These findings suggest that even spectatorship-passive consumption of sport-can generate psychological benefits when the team is perceived as actively supporting disaster recovery.

#### 4.1.3 Social reconnection as a proposed pathway

Disasters fragment social networks through displacement, bereavement, and destruction of community spaces (parks, gymnasiums, places of worship). Sport, particularly team-based and group activities, offers a structured mechanism for rebuilding social connections. Kunz's (8) evaluation of a sport and play program for traumatized children and youth following the 2003 Bam earthquake (Iran) provides rich qualitative evidence of social reconnection mechanisms. The program, running in two warehouses in refugee camps and a stadium in the nearby village of Baravat, engaged approximately 300 children aged 6–18 in football, volleyball, basketball, gymnastics, karate, and table tennis, led by locally recruited coaches. Over time, coaches observed that initially hostile and aggressive behaviors-typical reactions to trauma-gave way to cooperative team play, mutual support, and friendships that extended beyond the program. One coach reported: “We have developed a good team spirit so far and it is working well; there is a sense of team play while they are practicing and playing—it has improved a lot, maybe not a big change to last week, but much from the first months!” (8). The program also facilitated relationships between children and coaches as trusted adults; 74% of participants fully agreed with the statement “I usually share my private problems with my coach” (8). Notably, the most significant improvements were observed in participants who had experienced severe trauma, such as a girl who fell from a roof during the earthquake and suffered memory impairment. Through sustained participation and individualized coaching support, she became “one of our good players” with “lots of friends” (8).

Collective identity formation through sports spectatorship also contributes to social reconnection. Serazio (16) critically examined media coverage of the New Orleans Saints' unexpectedly successful 2006 performance following Hurricane Katrina. He argued that sports journalism invoked and negotiated the memory of Katrina, producing a largely uniform narrative that “relentlessly employed a winning team as the trope for metaphorical recovery and a means of the collective simultaneously coping with and escaping from traumatic memory” (16). The Superdome-a mnemonic site of trauma where thousands of refugees had sheltered during the hurricane-was “scrubbed away” and “exorcized of demons” through the Saints' home opener. While Serazio problematized this narrative as a substitute for real solutions (e.g., housing, healthcare), he acknowledged that for many fans, the team provided a genuine “weekly respite” and a sense of community solidarity (16). Similarly, Fortunato (15) examined the Florida Panthers‘ response to Hurricane Irma (September 2017) and found that the team's disaster relief efforts-including a 1 million donation, sheltering 2,500 relief workers at the BB&T Center, delivering 20,000 hot meals, and organizing supply drives-were perceived as authentic and credible because the team had a long-standing commitment to CSR and stakeholder relationships. The Panthers' campaign illustrated how sports organizations can leverage their community standing to mobilize resources and foster collective resilience (15).

### 4.2 Theoretical integration

These findings can be situated within extend existing theoretical frameworks in several respects. First, they support the integrated social identity model of stress, which proposes that social identification enhances wellbeing by providing access to social support (12). Inoue et al.'s (12) finding that team identification indirectly influenced community cohesion through emotional support (but not instrumental support) suggests that the perception of being cared for-rather than tangible assistance-is the active ingredient. This has implications for disaster response: sport organizations seeking to support recovery might prioritize symbolic actions (e.g., players visiting shelters, wearing memorial patches, holding moments of silence) alongside material aid. Serazio's (16) critique of the Saints' narrative as a substitute for real solutions notwithstanding, the psychological associations of such symbolic actions are empirically documented.

Second, the findings align with the social ecological model, which conceptualizes human development as nested within multiple environmental systems (microsystem, mesosystem, exosystem, macrosystem, chronosystem) (22). Domínguez and Yeh's (22) Social Justice Disaster Relief, Counseling, and Advocacy framework operationalizes this model by specifying interventions at the individual (e.g., crisis counseling, breathing techniques), interpersonal (e.g., support groups, storytelling circles), organizational (e.g., training volunteers, integrating mental health into school curricula), community (e.g., mobilizing resources, creating women-only spaces), and policy (e.g., advocating for equitable aid distribution, challenging discriminatory zoning laws) levels (22). Sport programs could be designed to target multiple levels simultaneously: for example, a community soccer league provides individual exercise (microsystem), team-based social support (mesosystem), opportunities for collective advocacy (macrosystem), and community rebuilding (exosystem).

Third, the review highlights the importance of distinguishing between different types of psychological distress. Hyland et al.'s (2) factor analysis demonstrated that peritraumatic dissociation, peritraumatic stress, anxiety/depression, and PTSD are separable constructs with distinct correlates. This suggests that interventions should be tailored to the specific symptom profile: for example, grounding techniques and mindfulness-based movement (e.g., yoga, tai chi) for dissociation; stress inoculation training and psychoeducation for peritraumatic stress; cognitive-behavioral therapy for anxiety/depression; and exposure therapy or EMDR for PTSD. Sport interventions that incorporate mindfulness elements may hold particular promise for dissociation, while competitive team sports might address social dysfunction. Nevertheless, these are theoretical extensions.

The findings suggest that sport-related activities may be associated with mental health benefits through multiple, interacting pathways. The conceptual distinction between participatory and spectator-based activities proposed in this review helps clarify a muddled literature. It implies that while direct participation might more strongly target physiological and behavioral pathways, spectator-based engagement leverages powerful socio-cognitive and collective identity mechanisms.

### 4.3 Methodological limitations and research gaps

Despite consistent positive effects, the evidence base has substantial limitations that must be acknowledged. First, it is critical to interpret the synthesized evidence within the context of significant methodological heterogeneity and limitations. The reviewed studies are predominantly observational, with designs including cross-sectional surveys (6), pre-post studies (7, 10), qualitative case studies (8, 14–16), and quantitative surveys (12, 17). These designs are suited to exploring associations, describing processes, and generating hypotheses. They are not designed to establish causality. Therefore, any suggestion that sport-related activities “improve,” “reduce,” or “facilitate” mental health outcomes should be understood as associations observed in the literature, not as confirmed effects. This is a primary limitation of the current evidence base.

Second, heterogeneity in intervention protocols, outcome measures, and follow-up periods precludes meta-analysis and limits generalizability. Studies used different definitions of “sport participation” (from daily unstructured play to weekly supervised exercise), different psychological instruments (e.g., GDS, IES-R, STAI, PCL-6, MMPI-A, ITQ), and different time points (1 month to 5 years post-disaster). This heterogeneity reflects the emergent nature of the field but complicates evidence synthesis and clinical guidance. This review formally addresses this heterogeneity by providing a *post-hoc* conceptual classification (participatory vs. spectator-based), but the effectiveness of each specific type of activity as an intervention remains to be empirically validated.

Third, we did not conduct a formal quality or risk-of-bias appraisal of the included studies. This is consistent with our narrative review design and its goal of conceptual synthesis, but it precludes any direct judgment about the internal validity of the contributing evidence. Positive findings should be weighed in light of potential selection bias, confounding factors (e.g., healthy volunteer bias, reverse causality), and other validity threats inherent in the designs of the primary studies.

Fourth, the search was limited to English-language publications, potentially excluding relevant studies in other languages.

Fifth, the physiological, psychological, and social mechanisms proposed here were rarely tested through formal mediation analysis within the primary studies. The pathways we describe, while grounded in the themes of the primary studies and supported by broader theory, are largely inferred from cross-sectional correlations or qualitative self-reports. Future research should incorporate direct measures of these mechanisms (e.g., cortisol, self-efficacy scales, social network analysis) to establish causal pathways.

### 4.4 Practical and policy implications

Despite these limitations, the consistency of positive associations across diverse settings, populations, and disaster types provides a sufficient rationale to consider sport-related activities as a promising element in post-disaster mental health strategies. Several cautious practical recommendations are proposed for further investigation and consideration:

First, disaster preparedness plans could include provisions for sports and physical activity programs, including pre-identified venues (school gymnasiums, community centers, places of worship), trained personnel (physical education teachers, coaches, community health workers, retired athletes), and equipment stocks (balls, mats, first-aid supplies, hygiene kits for women). The Rio Grande do Sul flood response, which mobilized 583 volunteers and 208 health professionals to distribute aid and provide care, demonstrates the scalability of community-based responses when infrastructure is in place (9).

Second, sports programs should be viewed as one component of multi-sectoral recovery efforts rather than implemented as standalone interventions. The Bam program's combination of sports, health education, conflict management, and violence prevention appeared more successful than sports alone (8). Domínguez and Yeh's (22) model integrated mental health counseling, case management, and material aid distribution with volunteer mobilization. This integrated approach addresses the reality that disaster survivors face multiple, interacting needs (food, water, shelter, health care, psychosocial support) that cannot be addressed in isolation.

Third, program design must be culturally adapted and community-led. Kunz (8) emphasized that local coaches were “more respected by the children and their parents: they knew their situation and shared the same experiences”. Domínguez and Yeh (22) argued that “effective disaster relief, counseling, and advocacy begin with a genuine belief that communities possess the strengths, knowledge, and agency to thrive despite the nature of the disaster”. Top-down programs imposed by external agencies risk being irrelevant, ineffective, or harmful. Culturally appropriate adaptations include gender-segregated sessions, female coaches, private facilities, flexible scheduling, childcare provision, and the integration of traditional games and rituals (e.g., indigenous sports, dance).

Fourth, female survivors warrant particular attention given their consistently higher reported trauma scores, unique physiological barriers (menstruation, pregnancy, breastfeeding), and social constraints (caregiving responsibilities, modesty concerns, safety fears) (6, 20). Strategies include women-only sessions, female coaches, private facilities with visual isolation (e.g., curtains, frosted glass), and flexible scheduling (e.g., evening hours after caregiving duties), and childcare provision (8, 20). These adaptations need not be expensive: simply reserving certain hours for women or repurposing underutilized spaces (e.g., building corridors, mezzanines, underground garages) can significantly increase female participation.

Fifth, sports programs may need to be sustained for months or years post-disaster, not just during the acute emergency phase. Hyland et al. (2) documented persistent distress 43 days post-earthquake, and Domínguez and Yeh (22) documented ongoing needs 3 months post-wildfires. Kino et al. (21) found persistent effects 5 years post-tsunami. Valenti et al. (7) detected significant changes 10 months post-earthquake. Short-term interventions (e.g., 6–12 weeks), while valuable, are insufficient for chronic or delayed-onset conditions. Donors and implementers should consider commit to multi-year funding and adaptive programming that evolves with recovery stages.

### 4.5 Strengths and limitations of this review

This review has several strengths. First, it offers a novel conceptual framework by formally distinguishing between participatory and spectator-based sport-related activities, bringing coherence to a fragmented literature. Second, it synthesizes evidence from diverse disciplines and cultural contexts (Asia, Europe, North America, South America, Middle East). Third, it employs a transparent thematic synthesis methodology with measures to enhance rigor, such as independent dual coding and consensus-based theme development. Fourth, it includes qualitative evidence that captures survivor voices and lived experiences.

However, this review has fundamental limitations, most notably its status as a narrative review. Without a systematic quality appraisal of the included studies or quantitative synthesis of effects, we cannot determine the strength of the evidence or the magnitude of any potential benefit. The findings are therefore exploratory and conceptual. They serve as a valuable starting point—coalescing disparate studies and generating testable hypotheses—but cannot serve as a basis for clinical or public health guidelines. Publication bias may also exist, as studies with null or negative findings are less likely to be published. Additionally, the absence of a formal risk-of-bias assessment and the reliance on self-reported outcomes in many studies introduce further uncertainty. The review should be seen as laying the necessary groundwork for future scoping and systematic reviews.

## 5 Conclusion

Natural disasters impose a substantial and sustained burden on mental health. Existing evidence, while methodologically heterogeneous and generally derived from observational study designs, indicates promising associations between engagement with sport-related activities and indicators of psychological recovery through proposed physiological, psychological, and social pathways. Structured, group-based, culturally adapted programs led by trained local facilitators appear most promising, but no comparative evidence establishes their superiority over other methods.

Given the early stage of research in this domain, these findings primarily serve a hypothesis-generating function. This review therefore does not advocate for the immediate large-scale implementation of sport-based interventions as evidence-based treatment. Rather, it argues for the recognition of sport-related activities as a prominent element within community-based recovery strategies that merits serious, rigorous investigation. Integration of sport into disaster preparedness plans should be considered cautiously and in tandem with multi-sectoral recovery efforts. Rigorous randomized controlled trials and systematic reviews (e.g., scoping reviews, meta-analyses of controlled trials) are urgently needed to determine causal effects before effectiveness claims can be made.
